# Bacterial community dynamics as a result of growth-yield trade-off and multispecies metabolic interactions toward understanding the gut biofilm niche

**DOI:** 10.1186/s12866-024-03566-0

**Published:** 2024-10-29

**Authors:** Amin Valiei, Andrew M. Dickson, Javad Aminian-Dehkordi, Mohammad R. K. Mofrad

**Affiliations:** 1 Molecular Cell Biomechanics Laboratory, Departments of Bioengineering and Mechanical Engineering, University of California, Berkeley, CA 94720 USA; 2 Molecular Biophysics and Integrative Bioimaging Division, Lawrence Berkeley National Lab, Berkeley, CA 94720 USA

**Keywords:** Microbial ecology, Social interactions, Altruism, Microbial community dynamics

## Abstract

Bacterial communities are ubiquitous, found in natural ecosystems, such as soil, and within living organisms, like the human microbiome. The dynamics of these communities in diverse environments depend on factors such as spatial features of the microbial niche, biochemical kinetics, and interactions among bacteria. Moreover, in many systems, bacterial communities are influenced by multiple physical mechanisms, such as mass transport and detachment forces. One example is gut mucosal communities, where dense, closely packed communities develop under the concurrent influence of nutrient transport from the lumen and fluid-mediated detachment of bacteria. In this study, we model a mucosal niche through a coupled agent-based and finite-volume modeling approach. This methodology enables us to model bacterial interactions affected by nutrient release from various sources while adjusting individual bacterial kinetics. We explored how the dispersion and abundance of bacteria are influenced by biochemical kinetics in different types of metabolic interactions, with a particular focus on the trade-off between growth rate and yield. Our findings demonstrate that in competitive scenarios, higher growth rates result in a larger share of the niche space. In contrast, growth yield plays a critical role in neutralism, commensalism, and mutualism interactions. When bacteria are introduced sequentially, they cause distinct spatiotemporal effects, such as deeper niche colonization in commensalism and mutualism scenarios driven by species intermixing effects, which are enhanced by high growth yields. Moreover, sub-ecosystem interactions dictate the dynamics of three-species communities, sometimes yielding unexpected outcomes. Competitive, fast-growing bacteria demonstrate robust colonization abilities, yet they face challenges in displacing established mutualistic systems. Bacteria that develop a cooperative relationship with existing species typically obtain niche residence, regardless of their growth rates, although higher growth yields significantly enhance their abundance. Our results underscore the importance of bacterial niche dynamics in shaping community properties and succession, highlighting a new approach to manipulating microbial systems.

**Supplementary Information:**

The online version contains supplementary material available at 10.1186/s12866-024-03566-0.

## Introduction

Bacterial communities are crucial components of various ecosystems, significantly contributing to the flow of energy and materials on the planet [1, 2]. They are present in diverse environments, such as the human body, where they form the microbiome—a determining factor in our health. A major portion of the human microbiome resides in the intestine, hosting a diverse array of bacterial species [3]. These communities enhance digestion, immunity, and overall gut function [4]. Complex microbial systems like the gut microbiome are typically highly dynamic, as community composition is affected by environmental conditions that vary with space and time [5, 6]. In the gut, for instance, many microbes live near the mucosa, forming a distinct sub-ecosystem adjacent to the epithelium, differing compositionally and structurally from luminal bacteria [7]. Temporally, mucosal communities are more durable and stable compared to luminal bacteria, playing pivotal roles in protecting the host tissue against pathogens and maintaining homeostasis [7, 8].

In understanding the differences in bacterial dynamics across ecosystems and sub-ecosystems, multiple factors need to be considered. One key factor shaping microbial dynamics is the nature of interactions among microbes, spanning various competitive and cooperative regimes [9]. While there is still significant debate about the dominance of each interaction for a specific environmental niche [9, 10], both types of interactions are present in numerous complex microbial ecosystems [9]. For example, it is increasingly evident in the gut that a diverse range of interactions exist within different individuals, which can yield diverse compositional outcomes [11, 12]. Besides the type of interactions, microbial characteristics and kinetic factors profoundly influence community dynamics [13, 14]. Both the kinetic rate of nutrient consumption and metabolic interactions determine bacterial abundance and positioning [13, 14]. Finally, the geometry and environmental properties of the system impact the diffusion of nutrients and significantly influence bacterial distribution [11, 13, 14].

Despite the multifaceted nature of bacterial community dynamics, existing techniques for studying these systems face significant challenges. For example, while luminal bacteria can be sampled through feces, directly observing the native microbial population within the intestine of live animals is notably difficult [15, 16]. While images of bacteria at cellular resolution in the intestine have allowed observation of the close-packed arrangement of hard-to-access mucosal communities [17], understanding how these microbial communities evolve over time and space remains unclear. This is because high-resolution real-time imaging in live animals is yet impractical [17, 18]. Moreover, there is insufficient data on the kinetic properties of individual microbes, as in-vitro measurements often differ significantly from in-vivo kinetic features [7]. The complexity of the involved variables and the lack of a fundamental understanding of mechanisms of microbial dynamics have further hindered progress in the field.

Recently, in-silico investigations using agent-based (or individual-based) modeling (ABM) have emerged as a powerful approach to deciphering microbial ecosystems and interpreting their dynamics. This approach, unlike traditional equation-based modeling, can capture the spatiotemporal subtleties of these systems. In ABM, the behavior of the system emerges from the collective actions of discrete agents with properties that can vary over time and space [19]. ABM is well-suited for predicting community structures based on metabolic microbial interactions in complex systems [20–22]. This approach can be utilized to assess the effects of parameters, such as the initial population composition, geometry, chemical interactions, and kinetics [20, 21] making it particularly valuable in modeling biofilm systems where bacteria are involved in strong localized interactions [20, 21].

Among the pioneering research on ABM of biofilms and bacterial communities is the landmark work of Kreft [23]. This study demonstrated that variations in kinetic parameters lead to intriguing dynamics among competing species [23]. Specifically, it showed that while species with faster growth rates are typically expected to outcompete slower-growing rivals [23], the dynamic shifts when slower-growing species have higher growth yields. In nutrient-limited environments such as biofilms, these slower-growing species can become competitive due to strong nutrient gradients and bacterial positioning [23].

Despite these insights, understanding microbial ecosystems, such as the gut microbiome, remains challenging due to the complexity of diverse interaction types, kinetic parameters, and temporal events, involving the introduction of newcomer species. In this study, our objective is to elucidate these effects with the overarching goal of dissecting the intricate communities living near the gut mucosa. We employed a model to simulate the dynamics of bacteria within a community of fixed thickness, constrained by the removal of the topmost biofilm layer. The composition of bacteria was determined by dynamic conceptual interactions, encompassing competition, neutralism, commensalism, and mutualism. We compared community properties with species having varying tendencies toward either a higher growth rate or a higher growth yield. Our findings highlight the intricate interplay between these effects, offering valuable insights into the dynamics of mucosal biofilms. Although our work does not provide a rigorous model of microbial dynamics in the gut due its complex physiology [7, 24], it contributes to a deeper understanding of microbial community behaviors and provides a foundation for future studies on the gut microbiome.

## Results

### Metabolic interaction and kinetics determine the outcome of two species communities

In microbial ecology, the spatiotemporal properties of communities, influenced by factors such as nutrient uptake rates and interspecies interactions, are highly important. Using an ABM, we investigate these properties in a simplified system resembling the geometry and microbial characteristics of gut mucosal bacterial communities. These communities are predominantly sessile and have heights ranging from a few tens to hundreds of micrometers above the mucosa [17]. They are subject to removal by fluid dynamics that can erode excessively tall bacterial structures from the topmost layer [17]. We simulate a 500 μm x 500 μm field around this niche, with nutrient diffusion from the top boundary driving bacterial growth, solved using the finite volume method (FVM). Growth is, however, restricted by colony size, with bacterial positions exceeding a predetermined height being removed from the system, similar to the approach adopted by Schluter et al. [25]. An agent-based description of each bacterium, including specific properties related to growth and replication, makes the model well-suited to simulating heterogeneous microbial populations. This allows us to capture the spatial and temporal dynamics of these communities, providing insights into their structure and behavior under various conditions.

In a complex system like the gut, an important element of heterogeneity is the metabolic features of bacteria. Regardless of their detailed metabolic pathways, bacteria generally exhibit either a high growth rate (*µ)* or high growth yield (*Y)*, as described by Monod kinetics [26], resulting in the well-known “growth-yield” trade-off [23, 27]. Bacteria with higher *µ* replicate faster, typically gaining an advantage in well-mixed, nutrient-rich environments [23]. In contrast, in niches with nutrient gradients, bacteria with higher *Y*—those more efficient at converting nutrients to biomass—can outcompete others, particularly in biofilm systems [23].

**Fig. 1:**
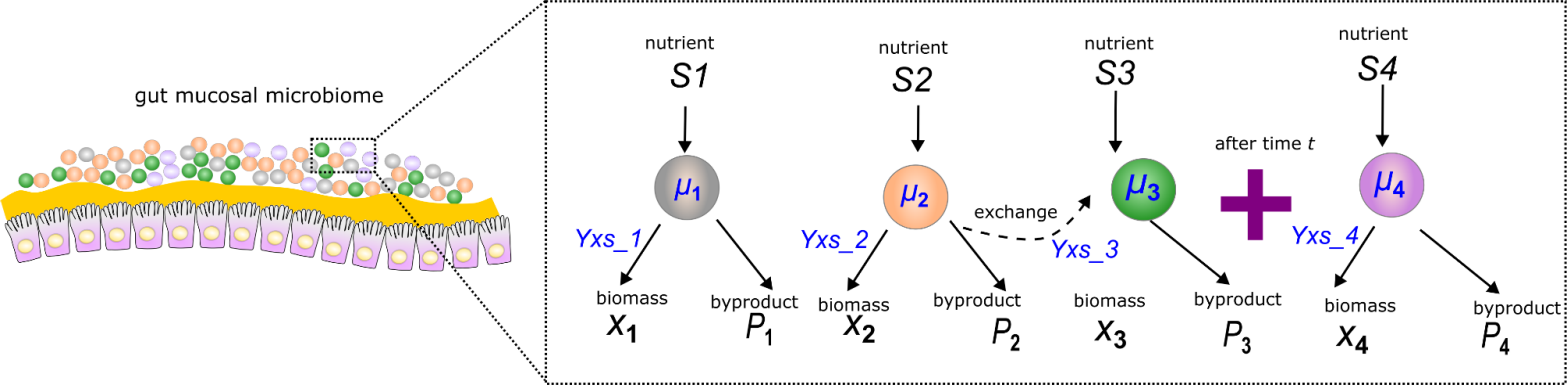
Schematic of a multispecies bacterial community inhabiting the gut mucosa. Interactions within these communities are typically complex, influenced by various interaction types and kinetic effects. Each bacterium may utilize different nutrients and has specific growth rates and yield coefficients, determining how quickly and efficiently each nutrient is converted into biomass. There may be exchanges between bacteria, where the metabolic byproducts of one bacterium is consumed by another. The community is also subject to temporal events; for example, additional bacteria may join an existing biofilm at any point during the community evolution

In a more complex system consisting of diverse interactions, chemical gradients arise from the diffusion of bulk nutrients and products, leading to various localized interactions, such as competition, neutralism, commensalism, and mutualism [24, 29]. These combined effects necessitate that our ABM model is set up to be flexible to handle multiple nutrient sources, chemical species, and multiple bacterial species that can enter the system at predefined times (Fig. 1). This allows the model to more accurately simulate the dynamic interactions and spatial organization of microbial communities in systems like the gut mucosa.

**Fig. 2:**
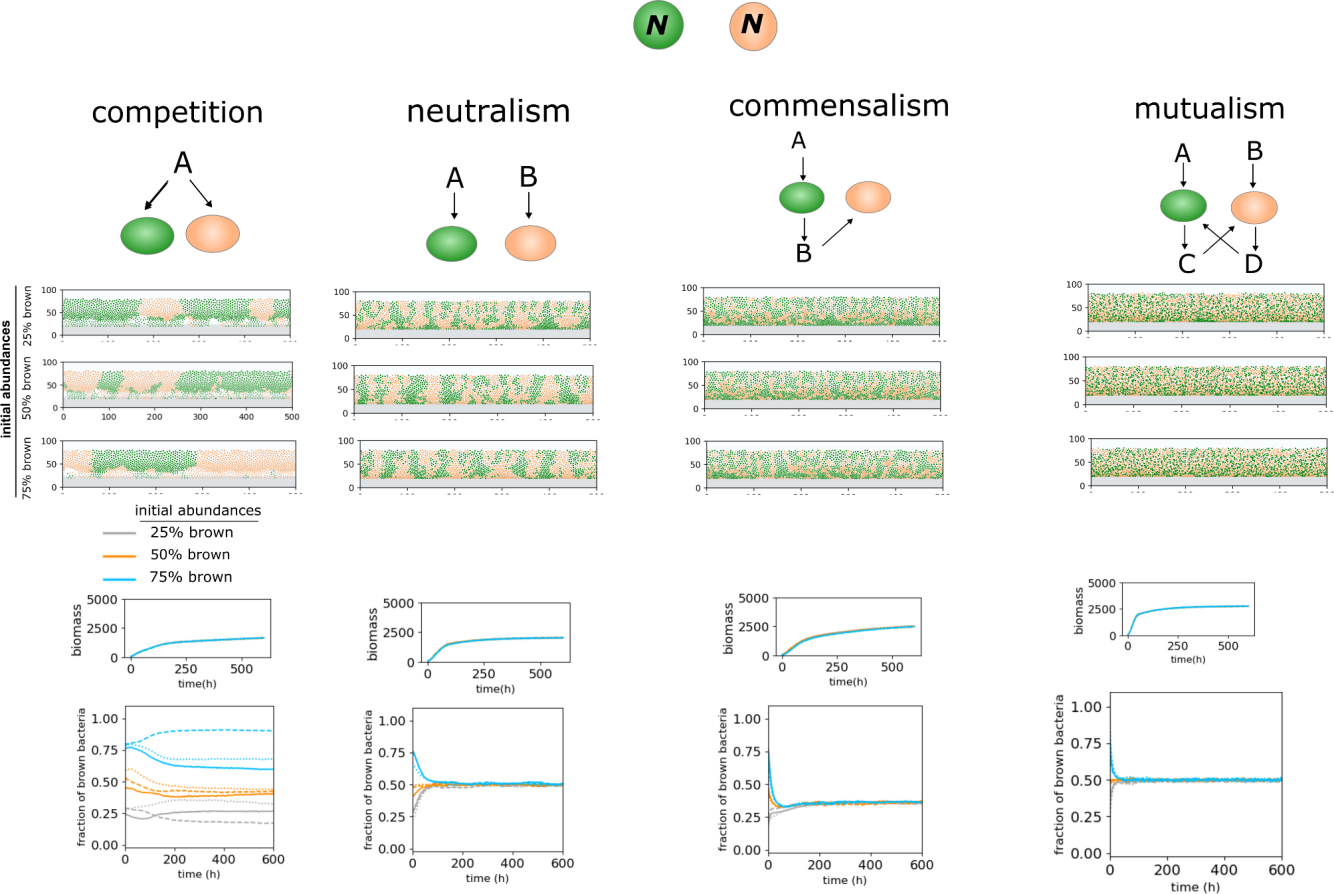
Bacterial community properties arising from metabolic interactions between two species. Modeling results include case studies for various interactions, including competition, neutralism, commensalism, and mutualism, each with three initial abundances. Both bacteria (labeled as N) have equal growth rates and growth yield coefficients. The model output, showing the side view of the community at a steady state, reveals morphological differences such as segregation tendency. Graphs show the total biomass amounts and biomass abundances over time for each scenario. In each graph, different initial abundance scenarios are represented by distinct colors, while repetitions are indicated by different line types (solid, dashed, and dotted)

We first investigate the effect of metabolic relationships on the colonization and growth of two species in a simplified scenario (Fig. 2). Each simulation begins with random colonization of species on the substratum and continues until the system reaches a steady state. In the competition scenario, bacteria directly interact to acquire the same bulk nutrient. Figure 2 shows that both species, having equal growth rates and yield coefficients, maintain compositions proportional to their initial abundance, forming competing clustered patches growing against the nutrient gradient. We define neutralism as a relationship where each bacterium type uptakes a distinct nutrient. With identical metabolic expressions, equal access to nutrients in this relationship promotes equal abundance and independent clustered colony growth (Fig. 2). We characterize commensalism as a relationship where one bacterial species uptakes the metabolic byproduct produced by the other, typically at a faster rate, equivalent to unidirectional cross-feeding. This scenario results in intermixing effects and balanced populations (Fig. 2). In bidirectional cross-feeding, also known as mutualism, both bacteria reciprocally consume the metabolic byproducts produced by one another. Mutualism leads to intense intermixing and population convergence (Fig. 2). The effect of these interaction types on population dynamics and morphological variation has been well-studied, and our results align with previous experimental and theoretical reports of similar structural and compositional properties for these interaction types observed in various contexts, such as radial microbial colony growth on surfaces and biofilm development on substrata [22, 28].

**Fig. 3:**
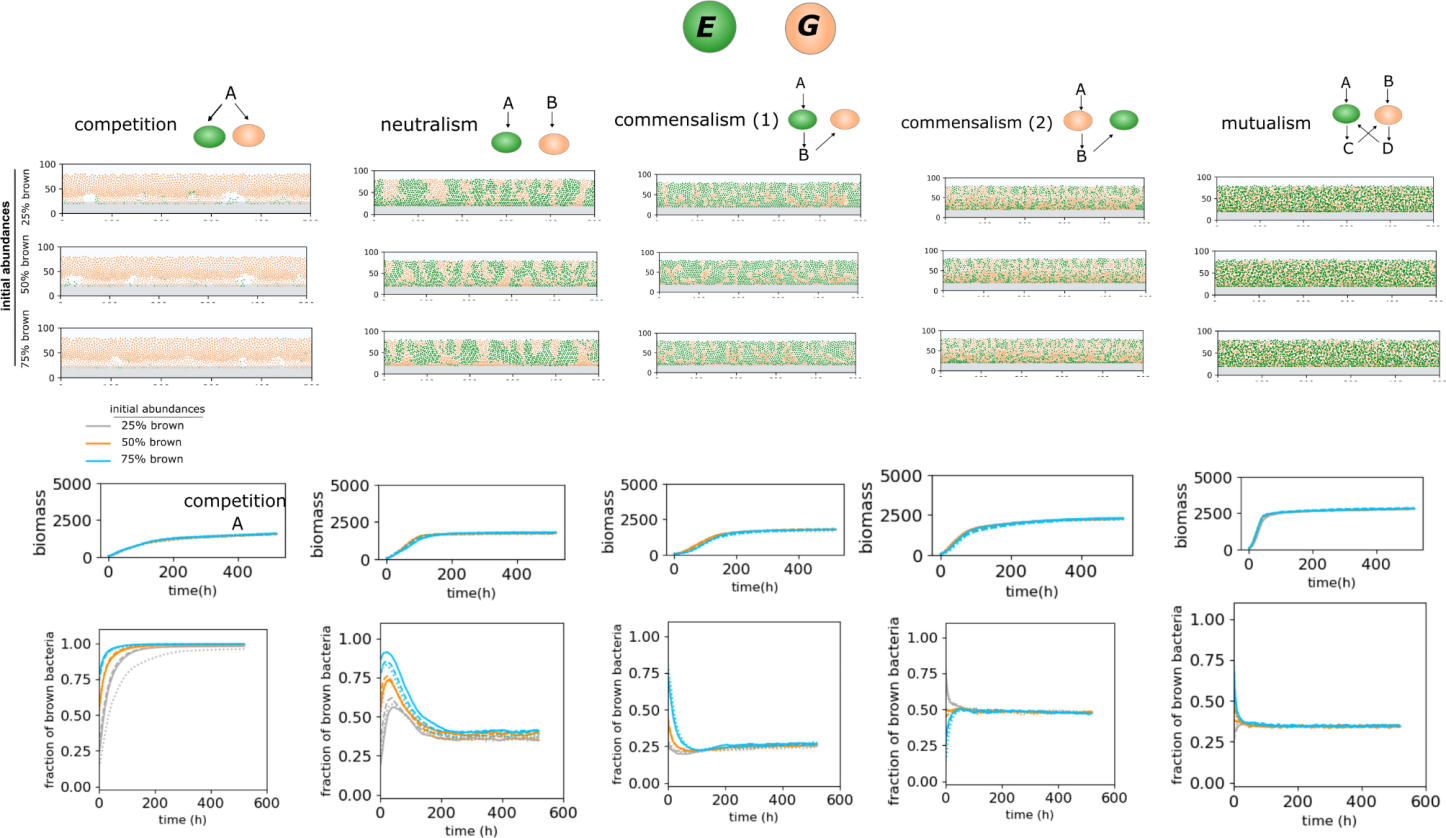
Bacterial community properties resulting from metabolic interactions between two species. Modeling shows results for various interactions, including competition, neutralism, commensalism, and mutualism, each with three initial abundances. Species have different growth rates and yield advantages; brown species have a higher growth rate (G), and green species have a larger growth yield (E)—refer to Table 1 for kinetic parameters. Simulation outputs display the side view of the community structure, and graphs show the total biomass amounts and biomass abundances over time for each scenario. In each graph, different initial abundance scenarios are represented by distinct colors, while repetitions are indicated by different line types (solid, dashed, and dotted)

Next, we examine the effects of kinetic features, notably the growth-yield trade-off. We repeated the simulations with a community consisting of species with different preferences for growth rate and growth yield (Fig. 3). In our simulations, one species has a higher growth rate, termed the fast-growing species (G), while the other has a higher growth yield, termed the resource-efficient species (E). Although the relationship between growth rate and growth yield are not always straightforward and predictable [30, 31], we assumed the product of both terms remains constant in the Monod equation to better understand the effect of growth rate-yield trade-offs (see Table 1), similar to Kreft’s assumption [23]. Results show that in competition, regardless of the initial composition, the faster-growing species dominate the community population (Fig. 3). As the community grows vertically, significant nutrient depletion suppresses the bacterial growth of the slower-growing species. In other interaction scenarios, both bacteria coexist with specific populational and morphological features (Fig. 3). In neutralism, even though both bacteria have equal access to nutrients, the fast-growing species eventually lose their abundance superiority to the resource-efficient species. In commensalism, the outcome depends on whether the faster-growing species is the beneficiary or the independent species. When the faster-growing species is independent, it gains a higher abundance; otherwise, it maintains a lower total population. In mutualism, the species with the higher growth yield consistently end up with a higher population than those with the higher growth rate. Importantly, in none of these non-competitive interactions is the species with the minor niche occupation totally suppressed. The morphological patterns differ across interaction types, with the highest clustering observed in competition, smaller clustering in neutralism, and high intermixing in commensalism and mutualism.

**Table 1:** Kinetic parameters for simulation of two-species bacterial communities

Species type	Bulk nutrient kinetics		Metabolic byproduct kinetics	
	µ (1/h)	Y	µ (1/h)	Y
Baseline species (N)	0.3	0.15	3	0.15
Fast-growing species (G)	0.6	0.15	6	0.15
Resource-efficient species (E)	0.3	0.3	3	0.3

These conceptual simulations demonstrate that the structural and populational community outcomes are strongly influenced by both the type of metabolic interrelationship and the kinetics. Bacteria with a higher growth yield or growth rate may dominate the system depending on the type of metabolic interaction. These results align with the idea put forward by Kreft [23] that faster-growing species do not always dominate bacterial communities.

### Metabolic interactions and kinetics impact community succession

Considering the significant impacts of metabolic features on community dynamics, the next question we pose is how the ecological outcome of a microbially-preoccupied niche is influenced by its prior state. While communities may develop through the co-colonization of multiple species, it frequently occurs that a mature biofilm encounters a new species that interacts metabolically with the existing community. One simplified scenario involves a niche initially occupied by one species, followed by the introduction of a second species after a delay. In our system, we simulate this effect by seeding five newcomer bacterium of a new species at midpoint heights after 300 h, into an existing mature biofilm and resuming the simulation until a steady state is reached. Considering that the newcomer species may adopt either a growth rate or a growth yield strategy, multiple scenarios are possible.

**Fig. 4:**
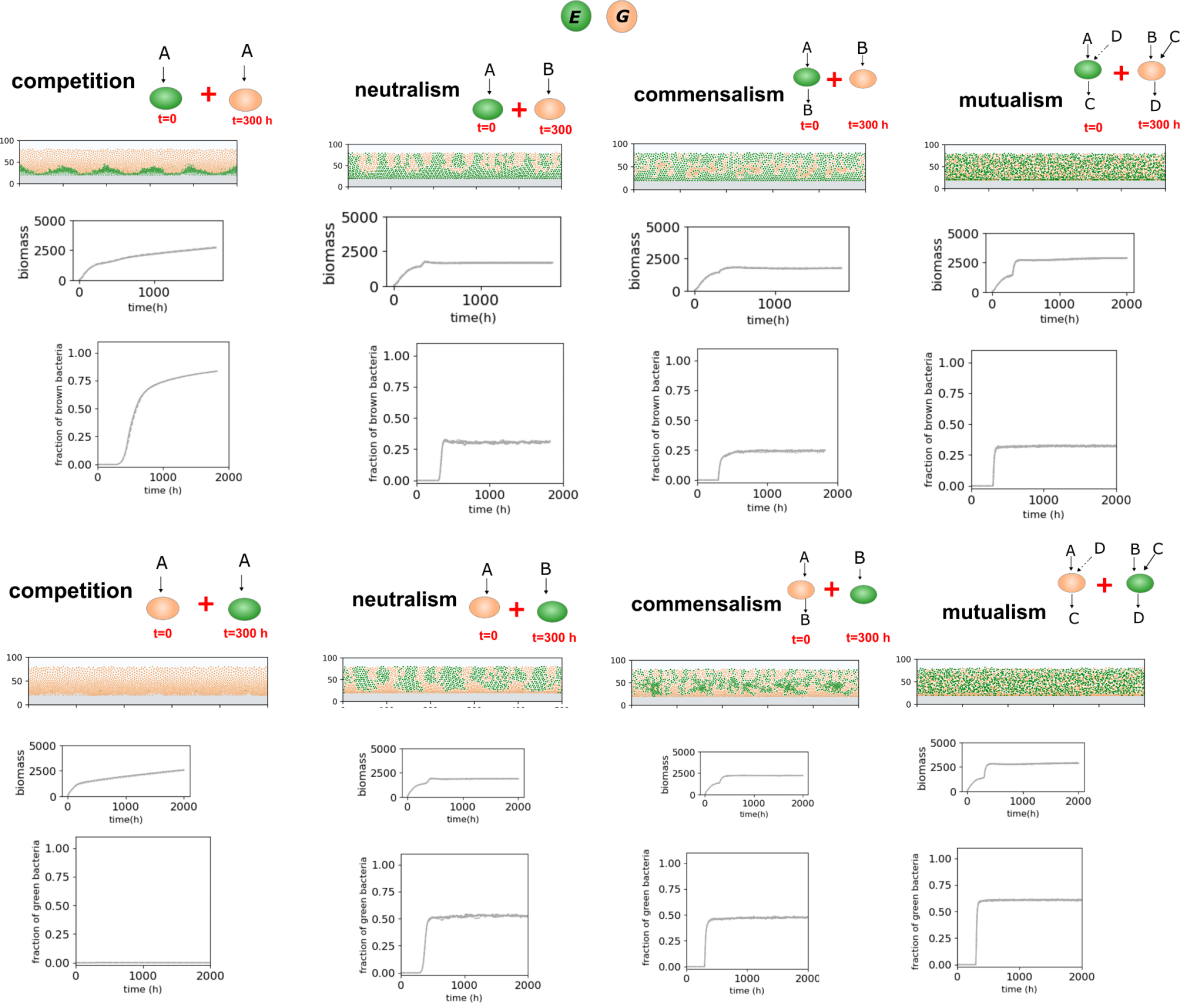
Community properties resulting from metabolic interactions, including the time-dependent introduction of bacteria. Modeling included case studies for various interactions, including competition, neutralism, commensalism, and mutualism, each with three initial abundances. Species have different growth rates and yield advantages; brown species (G) have a higher growth rate, and green species have a larger growth yield (E)—refer to Table 1 for kinetic parameters. The second species is added at mid-height after 300 h when the existing bacterial species have formed a mature, steady community. The model output shows the side view of the community structure, and graphs show the biomass levels and biomass abundances over time for each scenario. In each graph, repetitions are indicated by different line types (solid, dashed, and dotted)

When the pre-existing species consist of resource-efficient bacteria, a competing newcomer with a higher growth rate can gradually establish dominance in the niche, spatially outcompeting others (Fig. 4). This occurs because of increased nutrient consumption, which can “suffocate” the slower-growing species, pushing them away from the surface, as seen in previous reports [23]. In contrast, in non-competitive scenarios such as neutralism, commensalism, and mutualism, the resource-efficient species tend to achieve higher abundance over time (Fig. 4). Morphologically, in neutralism, although newcomer bacteria have access to an independent bulk nutritional source, they primarily develop in the upper half of the community, as downward growth is prevented by physical occupation and diffusion barriers. In commensalism and mutualism, newcomer bacteria, despite being introduced after a delay, disperse widely throughout the system, resulting in a relatively homogeneous and intermixed population (Fig. 4), similar to the patterns observed in co-colonization simulations.

When the existing population consists of faster-growing species, they maintain dominance, inhibiting the growth of the metabolically competing, resource-efficient bacteria. However, in neutralism, commensalism, and mutualism, the fast-growing species only gain a slight advantage from niche occupation, leading to a relatively balanced population as the community matures. Morphologically, we observe restricted and unidirectional dispersion of newcomers in neutralism, while both commensalism and mutualism exhibit significant interspersion. The observed dynamics reinforce the notion that competitive interactions are different from cooperative ones in various aspects. Strong cooperative interactions, regardless of the timing of species introduction, result in a larger niche occupancy and significantly impact community succession. Overall, it is the complex interplay between interaction types and kinetic features that determines both the population and morphological characteristics of the system as it develops over space and time.

### Metabolic interactions and kinetics determine the outcome in multispecies communities

**Fig. 5:**
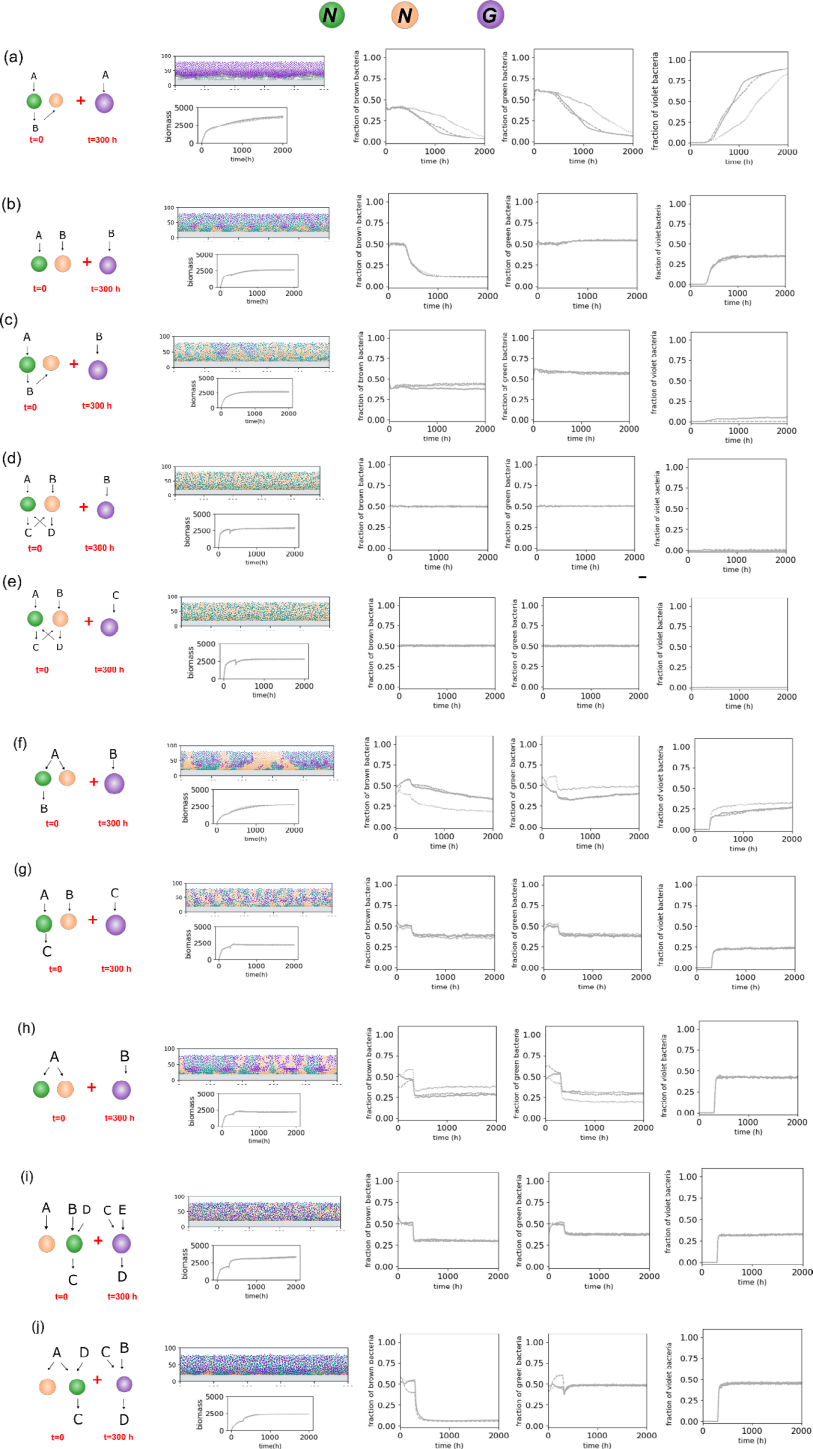
Community properties resulting from metabolic interactions among three species. Modeling included case studies for various interactions, including competition, neutralism, commensalism, and mutualism. The third bacteria (violet) enter the system at mid-height along the community at t = 300 h when a mature community has formed with green and brown species. The invading bacteria have a higher growth rate (G) than brown and green bacteria (N)- refer to Table 2 for kinetic variables. The model output shows the side view of the community structure, and graphs show the biomass level and biomass abundances over time for each scenario. In each graph, repetitions are indicated by different line types (solid, dashed, and dotted)

Having obtained basic insights into the dynamics of two-species communities, we now turn to the critical question of how microbial dynamics function in a system where various interaction types coexist. We present a model of a three-species community, allowing for different interactions among bacteria. Based on preceding results highlighting the contrast between competitive and non-competitive interactions—namely, neutralism, commensalism, or mutualism—we structure our discussion around this classification criterion. We investigate scenarios where the invading species has a higher growth rate (Fig. 5), kinetic features equal to those of the existing bacteria (Supplementary Figure S1), or a higher growth yield (Supplementary Figure S2)—refer to Table 2 for kinetic parameters.

**Table 2:** Kinetic parameters for simulation of three-species bacterial communities

Species type	Bulk nutrient kinetics		Metabolic byproduct kinetics	
	µ (1/h)	Y	µ (1/h)	Y
Baseline species (N)	0.6	0.15	6	0.15
Fast-growing invader (G)	1.2	0.15	12	0.15
Resource-efficient invader (E)	0.6	0.3	6	0.3

When a fast-growing species is introduced into a system with a commensal pair, it typically dominates the community (Fig. 5a). This aligns with the preceding findings, as the outcome is ultimately determined by the competition between the slow-growing independent species in commensal relationships and the fast-growing newcomers. Similarly, when a fast-growing species competes with a neutralistic pair (Fig. 5b), it exhibits substantial growth, displacing rivals from the upper biofilm section. However, when a faster-growing species competes over metabolic byproducts, it often loses to preexisting commensal species (Fig. 5c). In commensal relationships, the widespread dispersion of commensals occupying niches around the provider species inhibits newcomer establishment. This interesting dynamic highlights a clear distinction between competition over bulk nutrients and competition over metabolic byproducts. Resistance to colonization is highest when a faster-growing species competes with species in a mutualistic pair (Fig. 5d, e). In these scenarios, rapid growth and dispersion drive the suppression and expulsion of the newcomer species. Alternatively, in scenarios where the competitive species lacks a growth advantage (Supplementary Figures S1a-e and S2a-e), colonization by newcomers typically fails due to nutrient depletion, despite the fact that it remains possible if the competitive species exhibits a faster growth rate.

When a newly introduced species engages in commensalism with an existing competitive pair, it gains a foothold within the system, providing a competitive advantage to the species from which it derives nutrients (Fig. 5f). In contrast, in cases where existing species interact in neutralism, forming a commensal relationship with one party does not offer an advantage, as both existing species retain similar levels of abundance (Fig. 5g). In a competition-neutralism pair, the added neutralistic species strives to achieve a significant (∼ 50%) fraction of the population, merely hindered by limitations in nutrient availability within the niche (Fig. 5h). When the newcomer species forms a mutualistic relationship with the competitive pair, it displaces the non-mutualistic species from the system (Fig. 5i, j). Meanwhile, the mutualistic relationship between the introduced species and the existing community causes the dispersion of both species throughout the niche, leading to the complete elimination of the non-mutualistic species in a preexisting competition or the reduction of both existing species’ abundance interacting in neutralism (Fig. 5i, j). Comparatively, newcomers having equal or higher growth yields (Supplementary Figure S1f-j and S2f-j) can permanently colonize the system, with their ability depending on growth yield rather than growth rate.

Collectively, these data reveal essential patterns in resource and space sharing within densely packed communities. Firstly, the outcome of community composition depends on the type of interaction. Competition proves effective when it occurs at the appropriate trophic level, i.e., over bulk nutrients rather than metabolic byproducts. In competitive scenarios, the growth rate outweighs the growth yield, enabling faster-growing species to displace and eliminate slower-growing species. Neutralism ensures niche invasion and species coexistence unless one party has a sufficiently higher growth yield. Commensalism is multifaceted: it may confer spatial advantages to both the “provider” and the “beneficiary”, contingent upon the scenario. While the “beneficiary” reduces available niche space for the “provider”, it can provide the ecosystem with additional protection against newcomer invasion. Finally, mutualism is highly effective in deterring and displacing competing species. Even without a high growth rate, mutualism promotes fitness through emerging spatial effects involving intermixing and dispersion. Mutualism is likely the most beneficial and effective interaction in multispecies ecosystems when competing for survival against other sub-ecosystems.

## Discussion

Innately recognized as social entities, bacteria have long been observed to prefer living in aggregated structures that facilitate the exchange of nutrients and materials. Within complex communities, such as the gut, bacterial interactions are intricate, multi-layered, and interdependent. In the gut, for instance, specialized bacteria can digest diverse carbohydrates, including plant material, dietary fibers, bile acids, proteins, and other micronutrients [32, 33]. This results in resource partitioning, while concurrently, the multitude and diversity of bacterial species drive significant competition for nutrients [34]. Meanwhile, bacterial cross-feeding occurs through various intermediary nutrients, such as fermented metabolic byproducts [34]. For instance, acetate produced by certain species can be converted into short-chain fatty acids like butyrate by others [34]. These interactions result in a dynamic system rich in diverse ecological relationships within each niche, including competition, neutralism, commensalism, and mutualism [11, 35].

Previous studies have elucidated significant correlations between the spatiotemporal arrangement of microbial communities and their metabolic interactions [28, 36, 37]. It has been previously shown that competition leads to niche exclusion and clustering, whereas cross-feeding—seen in commensalism and mutualism—promotes species intermixing [22, 28]. This dynamic aligns with earlier findings in various contexts, such as radial expansion in colonies [28], biofilm growth on substrates [37, 38], and densely packed communities within the modeled gut mucosal niche [22]. Moreover, past research indicates that spatiotemporal structures, also influenced by nutrient gradients, are impacted by kinetic factors [23, 39]. Kreft’s influential work provides examples of such structures arising from the competition between growth rate and yield strategies [23]. While past studies underscore the importance of interactions and kinetics in shaping spatiotemporal patterns, the broader understanding of the combined effects of various factors remains incomplete.

In this work, leveraging an ABM framework, we explored the intricate relationships that may arise from the combination of kinetics and spatial effects originating from metabolic interactions. Our study differs from Kreft’s [23] work, which modeled unrestricted biofilm growth on a substrate, leading to tall, protruding biofilm structures and significant nutrient consumption. In that model, the concentration at the top boundary transiently decreases over time. By contrast, we assumed constrained biofilm growth, where bacteria above a certain height are removed from the system, allowing it to reach a steady state. We demonstrate that this difference in configuration leads to significant differences in results. While Kreft’s work suggests that, at sufficient initial densities, yield strategists may dominate toward the end of the growth phase, we found that rate strategists consistently outgrow others, regardless of initial densities. Furthermore, we identified dynamics arising from interactions beyond competition, which have not been addressed in Kreft’s paper. We observed that while competition drives clustered growth, cooperation through cross-feeding ensures species dispersion throughout a niche, aligning with earlier reports that highlight the significant impact of such metabolic interactions [23, 25, 28]. In terms of growth and yield trade-offs, we revealed that rapidly growing species may be outcompeted in neutralism, commensalism, and mutualism. The outcomes of these interactions largely hinge on the specific context and spatiotemporal conditions of the system. Notably, commensal interactions result in mutual benefits, such as enhanced ecosystem fitness, driven by spatiotemporal effects like species dispersion. Mutualism, in particular, stands out by significantly enhancing fitness across spatiotemporal scales. These results suggest that if altruism is defined more precisely in terms of commensalism and mutualism—rather than solely by growth yields, as in Kreft’s study [23]—it favors species with high growth yields over those with high growth rates.

Our findings have important implications for highlighting the effects of microbiome metabolic interactions in various contexts. For example, the gut mucosal microbiome is known to serve as a barrier against pathogens through a mechanism known as colonization resistance [7, 40]. This ability depends on the microbiome’s capacity to physically block pathogen penetration and inhibit their growth [7, 40]. Our findings demonstrate that the combined effects of kinetic factors and types of metabolic interactions within a mucosal microbiome substantially impact its ability to outcompete pathogens. They also highlight the importance of microbial mutualistic relationships, which are significantly more resistant to pathogen invasions due to their better niche fitness. Detailed analyses of metabolic patterns can serve as key in distinguishing a healthy microbiome from a dysbiotic one. Additionally, the success of microbiome therapies, such as fecal transplants, relies on the successful colonization of new species [41]. These interventions, while successful in many instances, have also been associated with noticeable failure rates [42]. We suggest that appropriate modeling of metabolic features can ensure the fitness of added consortia and the success of the treatment.

In summary, this work illuminates the intricacies of multispecies communities and presents a systematic bottom-up framework to unravel this complexity. Future models can be enhanced by incorporating metabolic networks and rigorous species metabolism modeling. Studies by Biggs et al. [43], Bauer et al. [44], and Scott Jr. et al. [45] have pioneered the integration of species-specific metabolic models into ABM models in research on microbial communities. With ongoing advancements in experimental models, such as in-vitro systems, acquiring kinetic data under in-vivo-like conditions will soon be feasible, which can enhance the accuracy of predictions. Such efforts are likely to yield models capable of deciphering microbial dynamics in the gut, addressing conditions that lead to imbalance and deterioration of diversity, and discovering new treatments to restore microbial balance. These advancements hold promise for significant contributions to understanding and managing human health.

## Conclusions

In this study, we simulated the spatiotemporal dynamics of multispecies microbial communities, focusing on their competition for, or sharing of, physical space and nutrient resources. Inspired by complex and densely packed microbial communities, such as gut mucosal communities, we imposed boundary conditions mimicking nutrient diffusion-driven growth while also considering constraints such as detachment due to fluidic effects. We employed an ABM approach combined with FVM, where bacteria were assigned predefined metabolic rules with varying kinetics and nutrient concentrations solved and tracked over time. Through multiple simulations, we highlighted the significant impact of kinetic factors and metabolic interactions on the spatiotemporal development of communities and their susceptibility to modification by newcomer species. We addressed the classic biochemical engineering dilemma of growth rate versus growth yield preference. Our findings indicate that competition favors species with higher growth rates, often resulting in the dominance of a single species. In contrast, cooperation or neutralism tends to support species with higher growth yields, leading to the coexistence of multiple species. Furthermore, we observed that transient events, such as the introduction of a newcomer species, are heavily influenced by the nature of interspecies interactions present. Species competing with higher growth yields are typically more likely to outcompete others for the same nutrients, although their population size is constrained by neutralism dynamics, wherein species with higher growth yields occupy larger spatial niches. In commensalism, beneficial species disperse more broadly across the niche, accessing deeper regions, while mutualistic bacteria create a highly resilient and competitive ecosystem, displacing and outgrowing non-mutualistic species. These foundational insights stress the importance of spatiotemporal dynamics in shaping microbial communities and highlight the necessity of incorporating such considerations into future ecosystem simulations.

## Methods

### Model overview

#### ABM general description

The agent-based model (ABM) represents bacteria as discrete spherical particles with variable mass, position, and species within a simulation field, similar to the previously-validated biofilm ABM models [46, 47]. The overall simulation is broken down into multiple modules that handle the physics within the system, including shoving and wall constraint, replication, and growth. The ABM also interacts concurrently with an FVM module, computing the concentration of all chemical species in the system. After initialization, a time process repeatedly steps the simulation forward by calling the physics and FVM modules in the appropriate sequence and updating agent states, concentrations of chemical states, and global parameters. Our ABM structure follows the approach in other key studies in this domain [23, 47]. Simulation parameters are summarized in Supplementary Table S1 and detail code implementation can be found in the Supplementary Information.

#### Agent properties

Each agent has a circular shape, representing a bacterium. The bacterial population at the start of the simulation is set to have an average mass of 1 unit, representing a bacterium with a diameter of 1 μm and a normal distribution with a 10% coefficient of variation and cut off outside two standard deviations [47]. Each bacterium has a continuous position with the 2-dimensional coordinates, and it has one of the two states of planktonic, representing bacteria in the liquid, which appear in the initial colonization phase (see below), or a frozen state, representing bacteria in the close-packed community. Some properties are specific to all bacteria belonging to the same species, such as kinetic parameters.

#### Model geometry and boundary conditions

The model geometry is a 2-dimensional box (500 μm × 500 μm) that simulates bacterial interactions in the system. It is chosen large enough to encompass system dynamics and transport processes involved. The bottom boundary is impermeable to bacteria. Any planktonic bacteria attached to the boundary will become frozen. The side boundaries are assigned free boundary conditions with a wrapped environment assumption, reflecting the periodicity of the system. The upper boundary is designated with a reflective boundary condition to prevent the escape of planktonic bacteria from the system when they reach this boundary. Given the model’s focus on the gut mucosal communities, similar to the approach followed by Schluter et al. [25], the height of the biofilm system is assumed to be constant. This reflects the idea that an excessively tall structure will be unstable and subject to removal by fluidic forces. Thus, any frozen bacterium will be removed above a specified height during biofilm growth (h = 60 μm above the substratum surface). The upper boundary is given a reflective boundary condition to avoid the escape of planktonic bacteria reaching the boundary from the system. FVM consists of a rectangular grid with 10 μm × 10 μm grid blocks. The boundary conditions for FVM are set to constant nutrient concentration at the top face, no flux at the substratum, and free boundary conditions at the side faces.

#### Simulation time advancement

The simulation runs in two distinct time phases. The first phase simulates bacterial colonization with smaller time steps (1 s). In this phase, a population of planktonic bacteria is injected into the system to create a community seeding layer resulting from their attachment to the substratum. The second phase has a longer time scale (15 min) for simulating community growth from the seeding layer. Planktonic bacteria are removed from the system at the end of the first phase. The simulation is resumed at least until the bacterial population achieves a steady state. In the simulations that involve the addition of a newcomer, the introduced species is injected after the existing bacterial population has achieved a steady state with each injection time explicitly stated in the manuscript text.

### Physical mechanisms

#### (ABM) Planktonic movement and bacterial colonization

Planktonic movement is a short, transient period at the beginning of the simulation intended merely to introduce randomly distributed colonization on the substratum for seeding biofilm growth. Planktonic bacteria are injected into the system with a random positional distribution between the bottom and the top boundaries. This module is thus only active for the colonization phase. It lasts for two minutes and results in the attachment of 50 bacteria to the system, forming the seeding layer from which biofilm grows. Bacteria in this phase are given a random walk motion with the following parameters: translational speed: 10 μm/s [48], rotational speed < θ>= ± 45° [49], and timestep: 1 s. Bacteria attaching to the substratum surface are turned into a frozen state. Following the colonization period, planktonic bacteria are removed from the system, and the simulation monitors the growth.

#### (ABM) bacterial attachment and wall constraint

Bacteria attach to the substratum any time they contact it. Attachment is detected through an algorithm whereby, for each bacterial movement, the position of the current and the next position are detected. If the future position falls inside the substratum boundary, the bacterial position follows a shorter displacement vector that avoids an intersection.

#### (FVM) concentration solution

We used FVM through FiPy package [50] to solve the concentration of all chemical species in the system. As the model is conceptual rather than accurate modeling of specific species and their metabolism, chemical species are defined symbolically based on the pseudo substances representing the properties of glucose as the model bulk carbon source and lactate as a model byproduct. Each chemical species in the system is within the grid block at position (x) by solving the following partial differential equation (PDE), ignoring the transient term within each time step:

1 $$\nabla .\left[ {{D_S}(Z).\nabla S(Z)} \right] + {R_S}(Z) = 0$$

In this equation, *S* is the concentration of the chemical species s, either nutrients or metabolites, and *D* is the corresponding diffusion coefficient. *R* is the biochemical reaction kinetics term accounting for all the consumption and production sources of *S*. The pseudo steady state definition of the PDE in Eq. 1 is based on the notion that chemical concentrations vary at a much faster time scale than the bacterial growth, like the approach proposed by Lardon et al. [47].; it saves computational time and simplifies the model structure. The diffusion coefficients for various pseudo substances are given in Supplementary Table S1. The growth yield and growth rate are two parameters that were varied during various case studies, which appear in the following extended form of R_s_ (z):

2 $$\begin{array}{*{20}{l}}{{R_{S_i}}(z) = - \sum\nolimits_{k = 1}^m {{\mu _{\max (k)}}\frac{{{S_i}}}{{{S_i} + {K_{s(i,k)}}}}{x_k}{Y_{{S_i}/{x_k}}}} }\\{ + \sum\nolimits_{k = 1}^m {\sum\nolimits_{j = 1}^n {{\mu _{\max }}_{(k)}} } \frac{{{S_j}}}{{{S_j} + {K_{s(j,k)}}}}{x_k}{Y_{{S_i}/{x_k}}}}\end{array}$$

This contains the dependence of the reaction rate on the bulk nutrient or metabolite concentration *S*_*i*_, with concentrations of biomass of each species *x*_*k*_ (*k* = 1, 2, …, *m*) in the grid block (Monod Kinetics). The consumption of a bulk nutrient or a metabolic byproduct *S*_*i*_ is reflected in the first term, and the generation of *S*_*i*_ from the cellular consumption of bulk nutrients or other metabolic byproducts *S*_*j*_ (*j* = 1,2, …, *n*) is captured in the second term. The Monod kinetics consists of the yield coefficient of the species *k* for nutrient *i*, termed $\:{Y}_{{S}_{i}/{x}_{k}}$, and the maximum specific growth rate $\:{\mu\:}_{\text{max}\left(k\right)}.$

#### (ABM) growth and division

After calculating the concentration of chemical species in each time step, the amount of biomass accumulation for each agent is calculated *via* Monod kinetics within grid blocks, following this equation:

3 $$\frac{{d{x_k}}}{{dt}} = (\sum\nolimits_{i = 1}^l {{\mu _{\max }}\frac{{{S_i}}}{{{S_i} + {K_{s(i, k)}}}} - m)} {x_k}$$

Once the biomass accumulation reaches a cut-off, the cell will split. If the unit of mass for an average new cell size is 1 unit, cells divide when they reach an average mass of 2 units. Cell division is set to have a Gaussian distribution with a 10% coefficient of variation and a cut-off outside two standard deviations [47]. The daughter cell is positioned in a random direction tangential to the parent cell.

#### (ABM) mechanical shoving

To address the issue of bacterial cell overlap caused by growth and replication, a shoving algorithm was used. The shoving algorithm calculates an overlap value for each cell pair by subtracting the center-to-center distance from the sum of radii. Displacement vectors were defined for each pair, oriented along the direction connecting their centers and equal to half the overlap distance. These vectors were used to compute new candidate positions for each cell simultaneously, ensuring that their movement avoids overlapping with neighboring cells.

## Electronic supplementary material

Below is the link to the electronic supplementary material.

Supplementary Material 1

## Data Availability

The datasets and simulation scripts generated during this study are available from the corresponding author upon reasonable request.
