# Supplementary material for: Bacterial community dynamics as a result of growth-yield trade-off and multispecies metabolic interactions toward understanding the gut biofilm niche
Source: BMC Microbiol. 2024 Oct 29;24:441. doi: 10.1186/s12866-024-03566-0 (PMC11523853; doi:10.1186/s12866-024-03566-0)
Supplement: Supplementary file 1 — Supplementary Material 1 [file 12866_2024_3566_MOESM1_ESM.pdf]

Supporting Information

Bacterial community dynamics as a result of growth-yield trade-off and multispecies metabolic interactions toward understanding the gut biofilm niche

Amin Valiei[1], Andrew Dickson[1], Javad Aminian-Dehkordi[1], Mohammad R.K. Mofrad[1,2*]

1. Molecular Cell Biomechanics Laboratory, Departments of Bioengineering and Mechanical Engineering, University of California, Berkeley, California 94720, USA

2. Molecular Biophysics and Integrative Bioimaging Division, Lawrence Berkeley National Lab, Berkeley, California 94720, USA

*Corresponding author: mofrad@berkeley.edu

Table S1. Simulation parameters

Parameter definition	Symbol	Unit	Value
Saturation constant	K_s	g.L^{-1}	0.2 (ref[1])
Maximum specific growth rate (baseline)	μ_{max}	h^{-1}	0.3 (ref[2])
Maintenance rate	m	h^{-1}	0.03 (ref[3])
Biomass yield coefficient (baseline)	$Y_{x/s}$	C-mol.C-mol^{-1}	0.15 (ref[2])
Product yield coefficient	$Y_{p/x}$	C-mol.C-mol^{-1}	2.5 (ref[2]) (based on lactate as an example metabolite)
Diffusivity of bulk nutrients	$D_{nutrient}$	m^2.s^{-1}	6.7×10^{-10} (ref[4])<br><br>(based on glucose as an example nutrient)
Diffusivity of metabolites	$D_{metabolite}$	m^2.s^{-1}	1×10^{-9} (ref[5])<br><br>(based on lactate as an example metabolite)
Bulk nutrient concentration	S_b	mM.L^{-1}	4 (ref[6])<br><br>(based on glucose)
Biomass molecular mass		g. C-mol^{-1}	27.8 (ref[2])
Bulk nutrient molecular mass		g. C-mol^{-1}	30.3 (glucose)
Metabolite molecular mass		g. C-mol^{-1}	30.0 (lactate)
Bacterial diameter		µm	1
Bacterial mass density		Kg.m^{-3}	1100 (ref[7])
Bacterial translational speed		µm.s^{-1}	10 (refs [8,9])
Bacterial rotational displacement		degree	45
Duration of the planktonic phase		s	100
Initial bacterial count (baseline simulation)			200
Simulation field size		µm× µm	500x500

Community height		60 μm
Simulation timestep interval (biofilm stage)	s	900
Number of invading bacteria		5
Height of the invading bacteria from substratum	μm	30

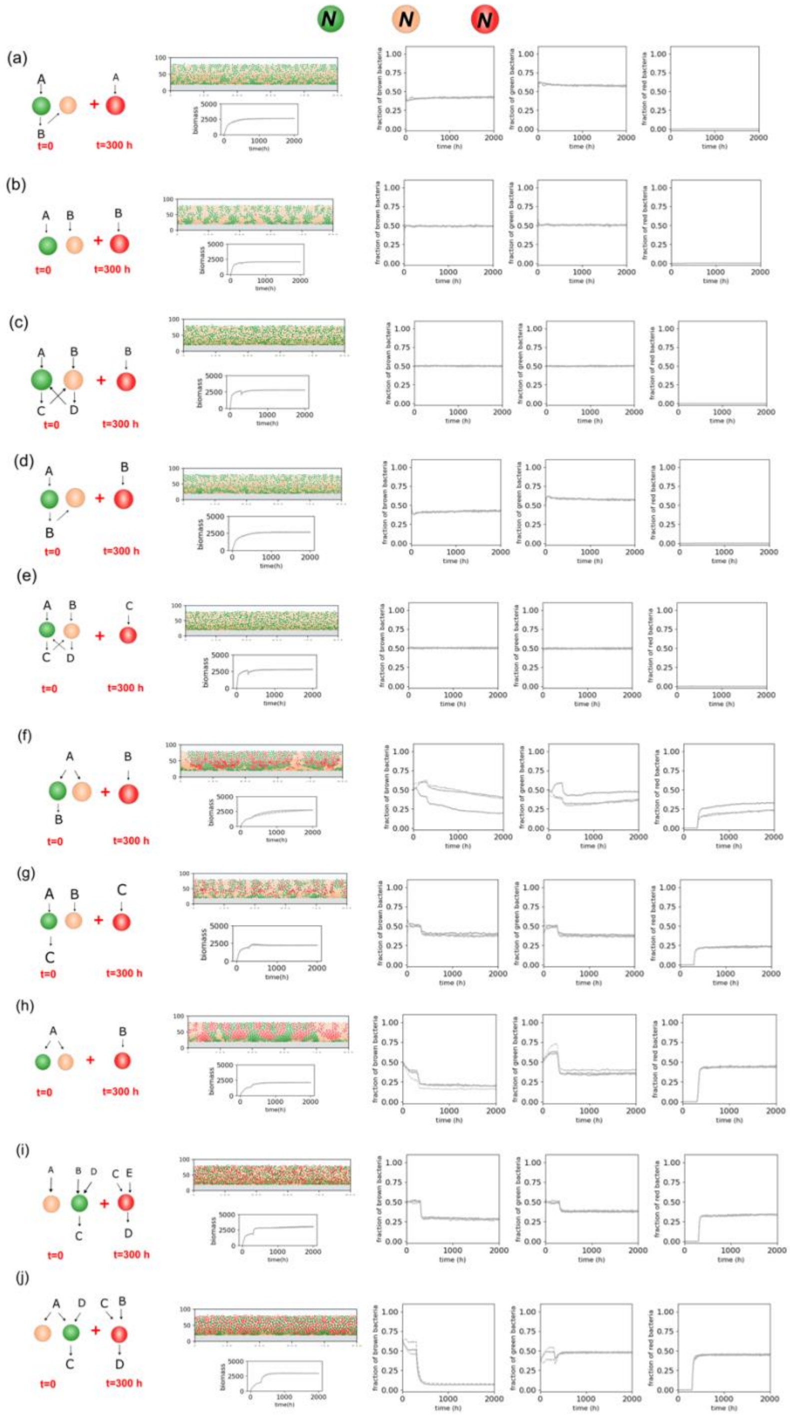

Figure S1. Community properties resulting from metabolic interactions among three spices. Modeling included case studies for various interactions, including competition, neutralism, commensalism, and mutualism. The third bacterial species (red) enter the system at t = 300 h, when a mature community has formed by green and brown species. The introduced bacteria (G) have a growth rate equal to brown and green bacteria (N)—refer to Table 2 for kinetic variables. The model output shows the side view of the community structure, and graphs show the biomass levels and biomass abundances with time for each scenario**.** In each graph, repetitions are indicated by different line types (solid, dashed, and dotted).

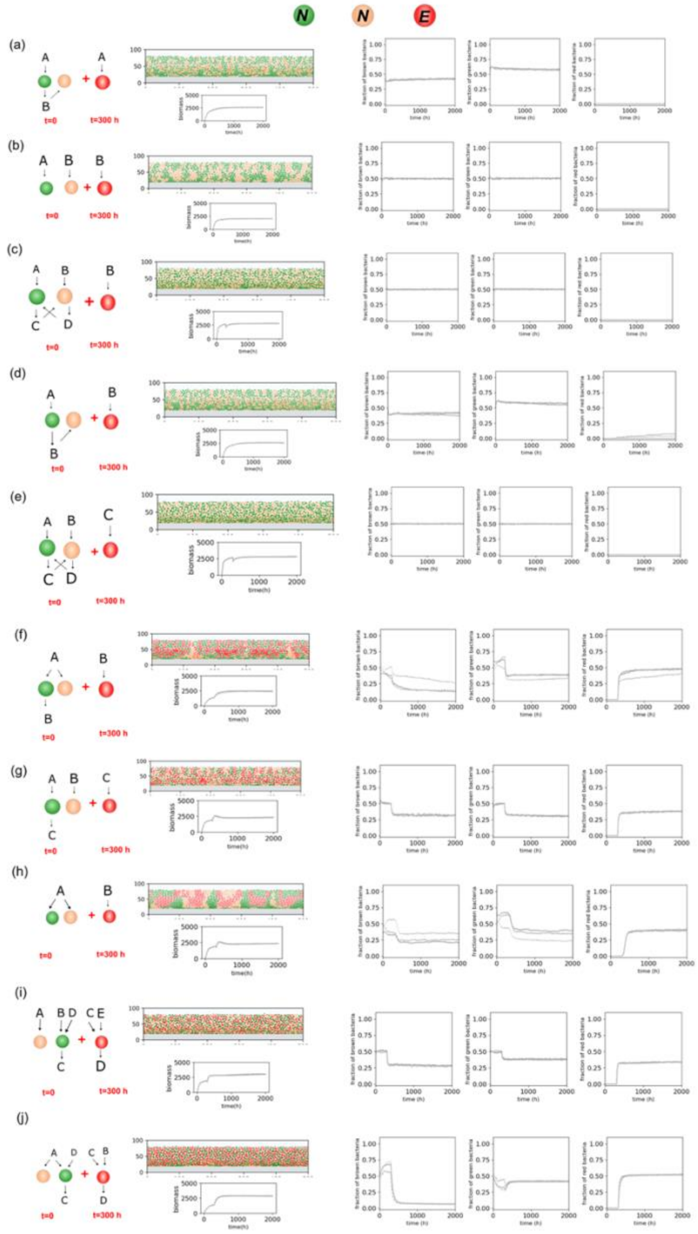

Figure S2. Community properties resulting from metabolic interactions among three spices. Modeling included case studies for various interactions, including competition, neutralism, commensalism, and mutualism. The third bacterial species enter the system at t = 300 h, when a mature community has formed by green and brown species. The introduced bacteria have a higher growth yield (E) than brown and green bacteria (N)—refer to Table 2 for kinetic variables. The model output shows the side view of the community structure, and graphs show the biomass levels and biomass abundances with time for each scenario**.** In each graph, repetitions are indicated by different line types (solid, dashed, and dotted).

ABM Code Implementation

Simulation Overview

The ABM consists of: (i) bacteria as the agents, whose information—such as mass, position, and species types—is stored in a NumPy array; (ii) a field class representing the geometry of the system, which is a 2D area of 500 µm × 500 µm. It stores key geometrical parameters, such as the presence of wall structures and the concentration of species again in NumPy arrays; (iii) functional modules, each responsible for simulating different processes, such as concentration calculation, collisions of bacterial cells with the wall and other bacteria, and replication and growth; and (iv) a timestep module in which the system events occur over time, where different functional modules are called, and agent and field states are updated.

1. Bacteria Agent Class

Bacteria are classified by species types, with each species having fixed properties such as growth rate and density. Comprehensive details of these simulation parameters are provided in Supplementary Table 1. Bacteria also have variable attributes, such as biofilm state (a Boolean flag), mass, and position. Additionally, bacteria have a variable called “next position” to manage collisions (see below) and an “existence” flag, which becomes false when bacteria are removed from the system (due to death or exceeding a certain height).

2. Field Class

Each field variable is represented as a 50×50 discretized matrix, with each element corresponding to a 10 µm × 10 µm area within the simulation field. Field variables are defined to represent geometry, including the presence or absence of walls (binary values), bacterial density (the total number of cells for each bacterial species), nutrient and metabolic concentrations, and diffusion coefficients in each grid block.

3. Functional Modules

3.1 Concentration Solver

The FiPy package is used to solve the reaction-diffusion differential equation in Equations 1 and 2 (manuscript text). It is based on the finite volume method applied to a discretized geometry. The meshed geometry is defined to be identical to the discretized geometry in the field class; that is, it is a 50×50 lattice representation with 10 µm × 10 µm grid blocks. One concentration variable is defined for each nutrient or metabolite in the simulation. The diffusion coefficients for each chemical species are provided in Supplementary Table 1.

We impose boundary conditions by constraining cell faces at the top of the volume to have a constant bulk concentration value; cell faces at the bottom to have zero flux, and side faces to have free boundary conditions. For substratum surfaces, diffusion coefficients are set to zero. Bacterial concentrations for a given species, as an input in the Monod model, are calculated within each finite volume block by summing the mass of each individual of that species within the block, normalizing by grid block volume, and storing the result in the corresponding entry of a discrete field. Field classes for diffusion coefficients and concentrations are transferred into FiPy mesh grids of equivalent dimensions.

Given the chemical boundary conditions and bacterial concentrations, the PDE representing chemical concentrations (Equation 1) within the biofilm system is formulated as a joint equation for bacterial feeding and metabolic by-products (Equation 2). The PDE is solved at each time step, after embedding the metabolic PDE into FiPy's data structures. The complete reaction-diffusion equation is described by using DiffusionTerm and ImplicitSourceTerm components, aligning with the discretized form of Equations 1 and 2.

The FiPy solver reduces the complex differential equations of the biofilm to a linear system on the grid. In the case of a highly non-linear system, the solver may not converge rapidly. To improve efficiency, the algorithm begins each PDE calculation with the solution from the previous time step, significantly speeding up the process through the sweep method.

3.2. Shoving Module

As the bacteria grow and replicate, a shoving algorithm is required to physically push them apart. The shoving algorithm is based on the iterative geometrical displacement of bacteria to avoid intersections. The algorithm iterates over each bacterium, identifying neighboring bacteria by finding those within a proximity of two bacterial maximal diameters. The center-to-center distances between the target bacterium and its neighbors are calculated, and intersecting pairs are identified—specifically, those with a smaller center-to-center distance than the sum of their radii. The intersecting pairs are symmetrically displaced by one-half of the overlap length (corresponding to a shoving factor of 1). Each shove occurs simultaneously for all involved bacteria, causing bacteria that overlap with multiple others to be pushed by the total displacement of all combined shoving vectors. Before the final position of the bacteria is implemented, it is evaluated for wall intersection using the wall constraint module.

3.3 Wall Constraint Module

Bacterial movements in each time step consist of the current position (output from the previous time step or t_0) and a new candidate position, calculated from shoving or planktonic movements. Before the new

candidate position is applied, it must be checked for intersection with the wall. Walls are defined as grid blocks that have a nonzero value in their field class matrix representation. To determine whether a bacterium intersects a wall, the integer part of the bacterial x and y positional indices is divided by the grid block size to identify the block where the bacterium is located. If the grid block is part of the wall, the point of intersection is calculated, and the bacterium's position is set to this intersection point.

4. Timestep Module

Once the simulation is initialized, functional modules are called sequentially to simulate biofilm development. The following events are included.

Creating a seeding layer

To simulate biofilm growth, a seeding layer of attached bacteria is required. Planktonic bacteria are introduced during a short initial phase to form this seeding layer. They freely move and attach to the substratum surface. A specified number of bacteria (200) are assigned random positions and directions above the surface within the simulation field. Bacterial positions are assumed to wrap around the environment, and the upper surface is reflective, corresponding to the condition where bacteria entering and exiting the system are equal.

At each time step, the position of the bacteria is calculated using the value from the previous time step and applying displacement, which is derived from translational velocity and a direction that changes in each time step (Supplementary Table 1). Bacteria become fixed when they contact a wall surface or another attached bacterium (biofilm flag turns on). The wall intersection module is activated to prevent collisions with walls, while the shoving module is used to avoid overlaps with other bacteria. This process results in the random attachment of bacteria to the substratum. Once the seeding layer reaches a population of nearly 50 bacteria, the planktonic phase ends, and all planktonic bacteria are removed from the system.

Biofilm development

The cell growth and division occur after the seeding layer is created. A zero value is assumed for solute concentrations, which are updated immediately at the first iteration through the concentration solver module given the upper boundary value, equal to the bulk concentration of solutes and the solid boundaries, at a no-flux boundary condition. The concentration solver receives bacterial mass density for each species in a grid block from the bacteria field and calculates the output concentration from the boundary conditions,

diffusion, and prescribed metabolic bioreactions. A timestep of 15 minutes is sufficient to simulate this process with enough accuracy.

Once the concentration of solutes is determined, bacterial mass accumulation is calculated by applying the nutrient consumption rate expression (Monod equation) multiplied by the biomass yield coefficient. A maintenance term per unit mass is then subtracted (Equation 3).

Once the new mass of bacteria is determined, the radii of the bacteria are updated. Bacteria with a mass greater than two divide, resulting in two bacteria occupying the same position. One of the bacteria is then moved away by a distance equal to its diameter in a random direction. A shoving module is applied to prevent the bacteria from overlapping, followed by a wall intersection module to avoid collisions with walls. Multiple iterations of shoving (approximately 10) result in the elimination of nearly all overlaps.

References

(1) Kovárová-Kovar, K.; Egli, T. Growth Kinetics of Suspended Microbial Cells: From Single-Substrate-Controlled Growth to Mixed-Substrate Kinetics. *Microbiology and Molecular Biology Reviews* **1998**, *62* (3), 646–666. https://doi.org/10.1128/mmbr.62.3.646-666.1998.

(2) Amaretti, A.; Bernardi, T.; Tamburini, E.; Zanoni, S.; Lomma, M.; Matteuzzi, D.; Rossi, M. Kinetics and Metabolism of Bifidobacterium Adolescentis MB 239 Growing on Glucose, Galactose, Lactose, and Galactooligosaccharides. *Applied and Environmental Microbiology* **2007**, *73* (11), 3637–3644. https://doi.org/10.1128/AEM.02914-06.

(3) Van Bodegom, P. Microbial Maintenance: A Critical Review on Its Quantification. *Microbial Ecology* **2007**, *53* (4), 513–523. https://doi.org/10.1007/s00248-006-9049-5.

(4) Kreft, M.; Lukšič, M.; Zorec, T. M.; Prebil, M.; Zorec, R. Diffusion of D-Glucose Measured in the Cytosol of a Single Astrocyte. *Cell Mol Life Sci* **2013**, *70* (8), 1483–1492. https://doi.org/10.1007/s00018-012-1219-7.

(5) Ribeiro, A. C. F.; Lobo, V. M. M.; Leaist, D. G.; Natividade, J. J. S.; Veríssimo, L. P.; Barros, M. C. F.; Cabral, A. M. T. D. P. V. Binary Diffusion Coefficients for Aqueous Solutions of Lactic Acid. *J Solution Chem* **2005**, *34* (9), 1009–1016. https://doi.org/10.1007/s10953-005-6987-3.

(6) Henson, M. A.; Phalak, P. Byproduct Cross Feeding and Community Stability in an in Silico Biofilm Model of the Gut Microbiome. *Processes* **2017**, *5* (1). https://doi.org/10.3390/pr5010013.

(7) Lewis, C. L.; Craig, C. C.; Senecal, A. G. Mass and Density Measurements of Live and Dead Gram-Negative and Gram-Positive Bacterial Populations. *Appl Environ Microbiol* **2014**, *80* (12), 3622–3631. https://doi.org/10.1128/AEM.00117-14.

(8) Saragosti, J.; Silberzan, P.; Buguin, A. Modeling E. Coli Tumbles by Rotational Diffusion. Implications for Chemotaxis. *PLoS ONE* **2012**, *7* (4), e35412. https://doi.org/10.1371/journal.pone.0035412.

(9) Cheong, F. C.; Wong, C. C.; Gao, Y.; Nai, M. H.; Cui, Y.; Park, S.; Kenney, L. J.; Lim, C. T. Rapid, High-Throughput Tracking of Bacterial Motility in 3D via Phase-Contrast Holographic Video Microscopy. *Biophys J* **2015**, *108* (5), 1248–1256. https://doi.org/10.1016/j.bpj.2015.01.018.
